# Mental health awareness, stigma, and help-seeking attitudes among Albanian university students in the Western Balkans: a qualitative study

**DOI:** 10.3389/fpubh.2024.1434389

**Published:** 2024-09-04

**Authors:** Zamira Hyseni Duraku, Holly Davis, Artë Blakaj, Arjeta Ahmedi Seferi, Klea Mullaj, Viola Greiçevci

**Affiliations:** ^1^Department of Psychology, University of Prishtina, Prishtinë, Kosovë; ^2^University Counseling Service, The University of Iowa, Iowa City, IA, United States; ^3^Ss. Cyril and Methodius University, Skopje, North Macedonia; ^4^Faculty of Social Sciences, Departament of Psychology, University of Tirana, Tirana, Albania

**Keywords:** mental health, higher education, students, socio-ecological model, Albanians, Western Balkans

## Abstract

**Introduction:**

The significance of mental health and its impact on overall well-being is increasingly acknowledged across various sectors, including higher education. However, despite this growing recognition, the stigma associated with mental health issues and related help-seeking, particularly within certain cultural groups and socio-economic contexts, continues to pose a formidable barrier to effective care, especially among university students. Addressing these challenges, this study explores the intricate interplay of factors affecting mental health awareness and stigma, including help-seeking behaviors among Albanian university students in three Western Balkan countries: Kosovo, North Macedonia, and Albania. By dissecting these multi-layered influences, this study aims to develop targeted interventions to enhance mental well-being and dismantle barriers to care in these regions.

**Methods:**

This qualitative study involved 60 Albanian university students from Kosovo, North Macedonia, and Albania, using focus groups to gather data. The discussions were structured around the socio-ecological model, facilitating a comprehensive exploration of the individual, interpersonal, organizational, and societal factors influencing mental health awareness, stigma, and help-seeking behaviors.

**Results:**

Findings from the study indicate a moderate awareness of mental health issues among students. Familial and cultural stigma among Albanians in the Balkans hinder open discussions and access to professional help. There is a notable lack of support from higher education institutions, with only 20% of students reporting that their mental health needs are met. These needs include affordable and confidential counseling, empathetic faculty interactions, and a supportive campus environment. Additionally, there is a lack of continuous and professional help within the community. Societal attitudes deeply rooted in Albanian cultural norms and traditional beliefs perpetuate stigma, limiting effective health care and help-seeking behavior.

**Discussion:**

The results emphasize the need for a culturally sensitive and holistic approach to mental health interventions that integrates strategies across multiple levels of the socio-ecological model. Enhancing mental health literacy, reducing stigma, and advocating for supportive legislation and policies in the Western Balkan region are critical. Moreover, the study highlights the urgent necessity for universities in particular to improve their mental health services, which will significantly enhance both the academic success and personal development of students.

## 1 Introduction

The importance of mental health and its consequential effects on overall well-being are increasingly acknowledged across various fields, including higher education (1). With the onset of the COVID-19 pandemic, global concerns regarding university students’ mental health have become even more pronounced. Many studies highlight major concerns, necessitating urgent responses. For example, according to the Healthy Minds Study (2), over 60% of college students internationally and in the United States reported at least one mental health disorder. Similarly, a study examining the mental health of higher education students in countries such as Poland, Slovenia, Czechia, Ukraine, Russia, Germany, Turkey, Israel, and Colombia indicated that approximately 60% of the sampled students showed symptoms of depression, generalized anxiety, or both (3). Increases in the prevalence rates of stress, anxiety, and depression among students have also been reported in studies conducted in the Western Balkans, particularly in Kosovo, Albania, and North Macedonia (4–7).

Given the developmental stage university students are in, which is the typical age of onset for many mental health disorders (8, 9), they are among the most at-risk groups for mental health challenges. This vulnerability is further compounded by significant barriers to seeking psychological help. These barriers include fear of self-disclosure (4), lengthy wait periods for mental health services, and doubts or misconceptions about the value, privacy, or accessibility of mental health care, all of which negatively impact students’ mental health and their attitudes towards seeking help (10). Additionally, the stigma associated with mental health and help-seeking behaviors continues to be a significant barrier for university students. Prior research indicates that this stigma can substantially affect population health (11), and is often more prominent at universities lacking stigma-reduction efforts and formal mental health services, such as the Western Balkans (4).

Cross-cultural and cross-country comparisons reveal meaningful differences in the level and impact of mental health stigma. For instance, Gallego et al. (12) found the highest levels of mental health stigma in Spain and the lowest in Canada. Socio-cultural factors not only influence individual stigma and help-seeking behaviors but also affect support networks, healthcare approaches, and community resources (13). These socio-cultural influences extend beyond stigma to broader aspects of students’ mental health and academic success. A large-scale study in Australia highlighted crucial connections between mental health and academic success for both domestic and international students, with international students being less informed about and less likely to use available health services both within and outside the university (14). Additionally, research in the UK identified enhanced mental health challenges faced by students of color and minority groups, among others, influenced by a lack of culturally competent support (15, 16). These findings underscore the need for significant research efforts to increase knowledge of mental health conditions and needs, including cultural considerations, to inform policies and service implementation to better support university students globally (17).

While such studies provide essential information about some countries and/or cultural groups, there is a notable dearth of literature that specifically addresses these dynamics within the Albanian populations, especially in the Western Balkans, particularly in the context of higher education and youth. This gap highlights a critical need for more research to understand the unique sociocultural dynamics that influence stigma and access to mental health services in higher education settings in this region and among these groups of populations. Therefore, against this backdrop, this study explored the perceptions of Albanian university students in Kosovo, Albania, and North Macedonia regarding mental health awareness and stigma and support services to foster a more comprehensive and nuanced understanding of the various factors that influence attitudes from the individual to the societal level in shaping help-seeking attitudes and experiences among Albanian students in the Western Balkan region. Indeed, understanding and addressing the multilayered factors that influence stigma is critical for improving care-seeking behavior and preventing mental health issues (13) for individuals in these countries and communities.

The participants in the current study were selected from three countries in the Western Balkan region of Southeastern Europe: Albania, Kosovo, and North Macedonia. Albanians constitute a significant proportion of the population in this region, with around 92% in Kosovo (18), nearly the entire population in Albania (19), and a substantial minority of around 24.3% in North Macedonia (20). These populations in close proximity to each other share language and cultural traditions, which may influence perceptions toward mental health. These countries also share similar complex sociopolitical histories that have had significant impacts on mental health systems and access, broadly affecting the entire population, not just university students.

For instance, Albania has been a closed country for 47 years owing to its dictatorial regime and communist ideology; this had significant impacts on citizens, especially in the 1990s, wherein several political and economic events occurred (21). Communism, as well as Soviet ideology, correspondingly impacted cultural attitudes and care for Albanians with mental disorders (22), and the nation is still attempting to recover in multiple health and economic spheres, including increasing the capacity for mental health services (23) and integrating training on mental health in the education of healthcare providers (24).

Similarly, Kosovo’s health system suffered as a result of violence and instability in the 1990s, particularly following the 1999 political upheaval, resulting in healthcare facilities being unable to provide basic health treatment. What did exist for mental healthcare was limited to inpatient or hospital-based neuropsychiatric care. In the early 2000s, Mental Health Reform focused on new strategies to shift limited existing services to community-based practitioners. It established a psychology department at the University of Prishtina as well as various professional associations and non-governmental organizations to reform the mental healthcare system (25). Despite these efforts, poverty and Kosovo’s post-war environment continue to contribute to social isolation and insufficient access to and funding for mental healthcare for university students and the general population, while a large proportion of the population experiences depression, anxiety, and post-traumatic stress disorders. Furthermore, mental health stigma and discrimination against people with mental health concerns remain widespread (4).

In North Macedonia, the collaboration between the World Health Organization (WHO) and the Ministry of Health from 2000 to 2008 marked a significant shift toward establishing mental health centers and advancing community-based care. This initiative was further supported by the National Strategy for the Promotion of Mental Health (2018–2025), which focused on improving access to mental health services and reducing stigma under the leadership of the Ministry of Health. Despite these developments, North Macedonia still faces hurdles in embedding comprehensive mental health support into its education system, underscored by a critical shortage of mental health professionals in schools and a lack of targeted mental health programs for youth (children through young/emerging adults). The youth in North Macedonia experience significant mental health issues, such as anxiety and depression. For example, in a recent survey by UNICEF, approximately one-third of the adolescent population reported symptoms of depression, and 42% reported experiencing moderate to severe anxiety. Despite these significant mental health challenges, more than half of the adolescents who acknowledge needing mental health services do not seek help, highlighting a substantial gap in support and accessibility (26).

Addressing mental health services for children, adolescents, and young adults is paramount, given the significant youth demographics in Albania, Kosovo, and North Macedonia. Kosovo has one of the youngest populations in Europe, with over half under 25 years of age, including 19% between 15 and 24 years. In North Macedonia, youths represent approximately 12% of its population of two million (27). Similarly, Albania’s youth demographic is fairly young, with youth under 15 years constituting 23% and those between 10 and 24 years constituting 21% of its population (28). However, the majority of universities in these countries do not offer any mental health services for their students, despite recent findings on the need (4, 29) and the positive impact of psychological services on academic growth, managing personal difficulties, and bettering emotional welfare (30).

Leveraging the socio-ecological model (31), the current study aimed to explore the differences and similarities in Albania, Kosovo, and North Macedonia at the individual (mental health awareness), interpersonal (family support, social networks, stigma), organizational (institutional responses, mental health service provision within universities), community (local support systems, community resources for mental health), and societal levels (laws, cultural norms, and societal stigma). The findings are intended to deepen the understanding of how these layered interactions contribute to or hinder mental health awareness and help-seeking behaviors among students. This comprehensive analysis will help in identifying targeted strategies for enhancing mental health support and reducing stigma within higher education settings in the Western Balkans and potentially in similar contexts globally. Developed by psychologist Urie Bronfenbrenner in the late 1970s (31), the socio-ecological model acknowledges that individual behavior is influenced by, and contributes to, multiple levels of the surrounding environment—from the immediate settings of family and school to broader societal and cultural contexts (32, 33). This model has proven effective in addressing various public health issues by identifying how layers of influence impact health behaviors and outcomes (34, 35).

The diverse cultural perspectives on mental health across the globe, affect the provisions, funding, and political support for such services, as well as university students’ willingness to seek help and their experiences when doing so (36, 37). Unsurprisingly, countries from economically developed regions have better-developed mental health resources, but unfortunately, much of Europe still has insufficient resources and governmental budgets for mental health (38), particularly the Balkan region (39). Therefore, the current study’s findings serve to increase such insights to better inform the development of culturally appropriate and responsive interventions in the Western Balkans, develop and update organizational policies, craft stigma-reduction narratives, normalize help-seeking, and facilitate continuous improvements and innovations in mental healthcare and support for university students.

## 2 Materials and methods

### 2.1 Study design

Our study employed a qualitative design to explore Albanian university students’ awareness of mental health and their perceptions of factors influencing stigma toward mental health and help-seeking behaviors in Albania, Kosovo, and North Macedonia.

### 2.2 Sample criteria and outreach

We recruited 60 university students from public and private institutions across Albania, Kosovo, and North Macedonia through purposive sampling (40, 41), aimed at capturing insightful contributions relevant to the research objective. Our focus was on students aged 18 years and older, currently enrolled in undergraduate or graduate programs, and self-identified as Albanian. Ensuring representation from various geographic areas, academic fields, and sexes was crucial.

Recognizing the importance of having the research informed not only by mental health experts but also directly by potential mental health service users (42), we focused on capturing the perspectives of Albanian students, a key demographic in our study. This approach has been a commonly-recognized inclusion in mental health research for the past 20 years (43).

Our recruitment began by leveraging existing networks and reaching out through direct contacts and collaborations with youth NGOs, university student associations, and academic forums. Additionally, we used a referral system (snowball sampling) (44, 45), encouraging initial respondents to share the study invitation with their peers and to inform us if we could contact them with more details. Following initial interest, personalized email invitations were sent to potential participants. These emails included detailed information about the study’s purpose, voluntary participation, confidentiality measures, and focus group schedules, ensuring informed consent (46).

### 2.3 Study setting

Data collection was conducted via online focus group discussions from January to March 2023, using virtual meeting platforms such as Zoom and Google Meet to accommodate participants from different locations, thereby eliminating travel barriers. We organized a total of six focus groups, each comprising 10 participants. Each session lasted approximately 60–90 min.

Focus group discussions were facilitated by psychologists and co-authors of this paper, who had undergone comprehensive training to facilitate the discussions effectively. The training, conducted by the senior researcher, also part of this research team, included a detailed manual that guided them on session structure and ethical considerations. Each moderator was also equipped with a structured tool of open-ended questions to delve into students’ perceptions of mental health and help-seeking behaviors. These questions were crafted to explore various levels of the socio-ecological model, from individual to societal influences. Furthermore, to reduce moderator bias as much as possible, we employed multiple research moderators in the focus group discussions. To uphold the highest ethical standards, our study adhered to stringent ethical standards in line with the 1964 Helsinki Declaration. At the beginning of each focus group discussion, all participants were re-informed about the study’s purpose and were provided with an informed consent form shared via Google Forms. Participants were asked to sign and submit the form, ensuring that they understood the study’s purpose and their rights, including confidentiality and voluntary participation terms. Additionally, participants were informed about, and they eventually consented to, the recording of the discussion, which was strictly used for data transcription, ensuring further accuracy in data handling and analysis.

### 2.4 Participant characteristics

The study included Albanian students from North Macedonia, Kosovo, and Albania, with each country contributing an equal proportion of 33.3% to the total sample size of 60 participants. Of the overall sample, 61.7 were women. Participants’ ages ranged from 18 to 26 years, with an average age of 21.1 years, and included students from various fields of study, including Medicine (28.33%), Social Sciences (28.33%), Education and Languages (13.33%), Economics (8.33%), Law and Arts (3.33%); they primarily resided in urban areas. Participating students were from 11 different universities located in the sampling countries, with 4 private and 7 public institutions. Participants from Albania were from the cities of Tirana, Gjirokaster, Fier, Lezhe, and Durrës. Participants were from the major regions in Kosovo, including the cities of Prishtinë, Gjakova, Pejë, Istog, Ferizaj, Gjilan, and Vushtrri. In North Macedonia, the study included participants from Tetovë/Tetovo, Skopje, Struga/Strugë, and Kičevo/Kërçovë. The majority of participants identified as Muslim (66.7%). For more detailed demographic information, refer to Table 1.

**Table 1:** Participant demographic information.

	*N*	%
North Macedonia	20	33.3%
Kosovo	20	33.3%
Albania	20	33.3%
**Sex**		
Male	23	38.3%
Female	37	61.7%
**Religion**		
Muslim	41	68.3%
Catholic	7	11.7%
Orthodox	4	6.7%
Atheist	2	3.3%
Agnostic	6	10.0%
**Sexual orientation**		
Heterosexual	47	78.3%
Homosexual	4	6.7%
Bisexual	1	1.7%
Preferred not to answer	8	13.4%
**Ethnicity**		
Albanian	60	100%
**Marital status**		
Single	54	90.0%
In a relationship	2	3.3%
Engaged	2	3.3%
Married	2	3.3%
**Field of study**		
Medicine	17	28.33%
Social sciences	17	28.33%
Education and languages	8	13.33%
Economics	5	8.33%
Law	4	6.67%
Computer science	3	5.0%
Computer Engineering	4	6.67%
Arts	2	3.33%
**Level of education**		
Undergraduates	50	83.3%
MA students	10	16.7%
**Residence**		
Urban	41	68.3%
Rural	19	31.7%
**Economic status**		
Average	48	80.0%
Middle	11	18.3%
Low	1	1.7%
**Employment status**		
Unemployed	44	73.3%
Employed	16	26.7%

### 2.5 Data analysis

The collected data from the focus groups were verbatim transcribed by the moderators, capturing all spoken words. To maintain confidentiality, participants’ names were replaced with pseudonyms, and any identifying information was removed. Each participant was assigned a code, which also included the moderator’s initials. These steps helped in re-reading the transcripts of the respective focus groups while listening to the recordings by another member of the research team, ensuring all data were accurately transcribed. The transcription of the focus group discussions was translated into English to accommodate the non-Albanian-speaking author. The final versions of the transcripts were then analyzed using a deductive thematic content analysis approach, guided by the socio-ecological model, to systematically explore the following predetermined themes: (1) Individual level: students’ mental health awareness; (2) interpersonal level: the role of families and friends in mental health support; (3) organizational level: university support and mental health infrastructure; (4) community level: engagement and resources in addressing mental health concerns; (5) societal level: legislations and socioeconomic and cultural factors influencing mental health attitudes.

Deductive analysis involves applying predetermined codes developed from organizational tools or theoretical concepts to the data (47). In this study, we used codes derived from the socio-ecological model to categorize data systematically, maintaining alignment with our research objective and applying theoretical constructs directly to the data (48).

To ensure the reliability of our findings, multiple researchers independently coded the data using NVivo 14 software, which facilitated the systematic organization and analysis of the content. After the independent coding phase, all coding outcomes were consolidated and discrepancies were discussed and resolved in team meetings. This collaborative review process helped to ensure consistency and objectivity in data interpretation. The analysis achieved a reliability score of 89%. During the analysis process, we monitored for data saturation, which was reached when no new information emerged. This point was initially identified after analyzing data from the first four focus groups. To confirm saturation and ensure comprehensive coverage of diverse perspectives across the study regions, we conducted two additional focus groups.

## 3 Results

### 3.1 Individual level: students’ mental health awareness

Increased mental health awareness among students was commonly reported, attributed to various educational programs. These included lectures and workshops, which were conducted within their education institutions, and in many cases organized by local mental health NGOs aimed at improving citizens well-being. Approximately 35% of participants from all three countries reported a great understanding of mental health issues following these interventions.

“The stress management workshop organized by our university helped me realize how stress affects my studies and personal life.” (Participant from Kosovo, aged 22 years).

“I learned about emotional resilience and techniques for managing anxiety through workshops held at my school, organized by a local NGO.” (Participant from North Macedonia, aged 20 years).

“Our university provided a series of mental health basics that opened my eye to the importance of mental health.” (Participant from Albania, aged 23 years).

In general, participants from the three countries reported similar engagement in mental health education. However, while referring to educational mental health awareness programs, participants from North Macedonia, highlighted a notable lack of awareness and fewer organized interventions. This gap in mental health initiatives could be owing to budget constraints or fewer collaborations between universities and mental health organizations, as participants stated.

### 3.2 Interpersonal level: the role of families and friends in mental health support

Approximately 70% of students highlighted the critical role that family and friends play in their mental health support. However, the experiences vary considerably, with some students receiving substantial support, while others face challenges owing to cultural stigmas influencing their peers’ willingness to discuss mental health issues, as well as generational misunderstandings within their families.

“While my immediate family is supportive, taking active steps like consulting mental health professionals, the extended family and broader social circle sometimes express harsh views, showing a significant gap in understanding.” (Participant from Kosovo, aged 22 years).

“I rely on my friends for support, but there’s still some hesitation to talk openly about deeper issues. My family does not really ‘get’ mental health, so it’s not something we discuss much at home.” (Participant from North Macedonia, aged 21 years).

“There’s support from my family, but also some generational misunderstanding about mental health. It’s like they try to understand, but they also hold onto traditional beliefs that mental health issues can just be ‘overcome’ with willpower.” (Participant from Albania, aged 25 years).

In Kosovo, immediate family members often provide essential support and undertake efforts to better understand and manage mental health conditions. However, extended family and broader social networks sometimes hold stigmatizing attitudes. The prevalence of more stigmatized views among older generations within families is a common issue highlighted across participants from North Macedonia and Albania, and Kosovo. This influences the youth and poses challenges for students seeking support within their families or among friends.

### 3.3 Organizational level: university support and mental health infrastructure

The support provided by universities in Kosovo, North Macedonia, and Albania for mental health reveals a complex landscape of limited resources and varying degrees of faculty involvement. Approximately 20% of students reported that their mental health needs (as self-assessed by the participants) are adequately addressed by university services, highlighting a broad deficiency in support across these regions. These needs primarily include access to affordable and confidential counseling services, understanding and empathetic faculty interactions, and a supportive campus environment that mitigates academic stress. The deficiency in support is exacerbated by stigma around mental health issues within their educational institutions. Additionally, the dismissal of stress and anxiety by faculty members contributes to students feeling unsupported, as professors often dismiss their struggles.

In Kosovo, although some students benefit from mutual support groups, the overall availability of counseling services is notably limited. The majority of students pointed out that they have never encountered any mental health support services at their universities. In North Macedonia, the scarcity of mental health resources is equally stark, with only minimal counseling services available. In Albania, the sporadic nature of mental health support through workshops proved insufficient for continues support.

“Our campus does not have counseling services; however, I have benefited from peer support services (mutual support groups) in my department.” (Participant from Kosovo, aged 23 years).

“There’s one counselor for the whole university, which is nowhere near enough.” (Participant from North Macedonia, aged 22 years).

“Sometimes there are workshops, but regular, ongoing support is lacking.” (Participant from Albania, aged 26 years).

In Kosovo, a noteworthy portion of students expressed concerns about the lack of support from their professors. Similarly, Albania and North Macedonia face challenges in faculty engagement with their students’ socioemotional well-being. Participants highlighted the lack of holistic approached in universities, the lack of empathy toward students’ academic stress, and the discouraging academic environment created by faculty members.

“…I think that universities should take into consideration our mental health. I believe that professors could have been a little more understanding, for example, to know that besides academic responsibilities, we are also people, and we also have our own personal lives.” (Participant from Kosovo, aged 24 years).

“The treatment from professors has a very negative impact.” (Participant from North Macedonia, aged 23 years).

“‘You will not have it easy with me; most fail.’ This was the first lesson we had in our first lecture at the university; in all that university experience, you expect to have at least a nice a warm welcome because it’s a big transition…and this was, this was the first sentence we were told.” (Participant from Albania, aged 21 years).

Across all three countries, the feedback from students highlights noteworthily gaps in the availability of mental health services within their universities. The lack of faculty engagement and understanding further exacerbates their challenges, creating an environment where students’ mental health needs are neither fully understood nor adequately addressed. Additionally, the fear of being judged or misunderstood by both peers and faculty members adds to the reluctance to address mental health concerns openly.

### 3.4 Community level: engagement and resources in addressing mental health concerns

Community support for mental health across Kosovo, North Macedonia, and Albania displays a complex picture of engagement, with varying levels of service availability and quality. Approximately 40% of participants across the three countries report positive community engagement in mental health discussions. However, this engagement often lacks consistency and is impeded by insufficient support and resources.

In Kosovo, there is a noticeable trend toward increasing openness in discussing mental health, particularly among younger demographics. Participants note a growing awareness but also highlight a critical gap in professional services to meet community needs. In North Macedonia, while there are some community initiatives and campaigns for mental health awareness, participants describe these efforts as sporadic and often poorly funded, leading to a fragmented support system. Furthermore, stigma toward seeking help prevalent within the community also makes people hesitant to seek help. Similarly, in Albania, the stigma around discussing mental health remains strong, with many such topics of discussion still considered taboo within communities. This stigma is compounded by a lack of funding and resources for mental health services, making effective support challenging to sustain. Lack of trust and the skepticism toward the quality of private mental health services is also highlighted as a specific challenge within the communities from both Kosovo and Albanian participants.

“In my town, there’s a growing openness to discussing mental health, but professional services to meet the demand are lacking.” (Participant from Kosovo, aged 21 years).

“Community initiatives are there, but they are not consistent, and people often do not know where to turn for help.” (Participant from North Macedonia, aged 23 years).

“There’s a slow improvement in community understanding, but the available services are far from adequate and stigma remains high.” (Participant from Albania, aged 24 years).

Overall, the level of community engagement and resource availability in these countries reflects a broader regional challenge of addressing mental health concerns. While Kosovo shows some progress in reducing stigma and improving openness, the actual availability of mental health services still falls short of community needs. In North Macedonia and Albania, the intermittent nature of mental health initiatives and the pervasive stigma around mental health discussions hinder effective community support.

### 3.5 Societal level: legislations and socioeconomic and cultural factors influencing mental health attitudes

In Kosovo, North Macedonia, and Albania, societal attitudes toward mental health are deeply entrenched in the cultural context and social norms that often view mental health issues through a lens of stigma and secrecy.

Kosovo is seeing gradual changes toward openness in discussing mental health, especially among urban youth, who are slowly challenging traditional taboos surrounding mental health. Despite these changes, broader societal acceptance remains limited, with mental health issues often not discussed openly owing to fear of social rejection or misunderstanding. North Macedonia exhibits a strong cultural inclination toward privacy and self-reliance, where mental struggles are considered personal weakness rather than health issues. In Albania, traditional beliefs strongly influence public perception, often interpreting mental health struggles as a source of shame for individuals and their families, which discourages people from seeking or offering support.

The absence of institutional support and comprehensive mental health policies further complicates the landscape of mental health care in these countries. In Kosovo, while there are some initiatives aimed at improving mental health awareness and support, they often lack coordination and governmental backing, which diminishes their potential impact. In North Macedonia mental health services are scare and poorly integrated into the health system, reflecting a broader governmental oversight in prioritizing mental health as a public health issue. In Albania, the absence of proactive public health strategy for mental health means that services are sporadic and underfunded, relying heavily on non-governmental organizations and private initiatives that cannot comprehensively address the needs of the population.

“There’s growing talk about mental health, but it’s still a sensitive topic for many. We need more than just talk; we need action and accessible services.’ (Participant from Kosovo, aged 22 years).

“Seeking mental health support is seen as a weakness. Our society values toughness, which is a barrier to mental health care.” (Participant from North Macedonia, aged 24 years).

“In our society, admitting you are seeing a psychologist is like saying you have a serious defect. It’s very discouraging. The Ministry of Health needs to exert some influence or engage in collective work to first make people aware and inform them about what mental health truly is.” (Participant from Albania, aged 23 years).

These insights highlight the complex interplay of cultural attitudes, institutional shortcomings, and the pervasive impact of stigma on mental health across Kosovo, North Macedonia, and Albania.

## 4 Discussion

Our study findings reveal complex interactions across individual, interpersonal, organizational, community, and societal levels that influence mental health stigma. Examined through the socio-ecological model, these findings highlight how influences at one level affect, and are affected by, other levels.

At the individual level, mental health awareness often involves barriers owing to stigmatizing attitudes within interpersonal relationships, particularly familial contexts. This aligns with findings in Europe and globally (13). In the Western Balkans, educational interventions aimed at increasing individual awareness often align with traditional views that undermine these gains. Furthermore, despite the influence of cultural stigma on awareness, it also affects the level of support students receive from family and peers. As noted by the participants, cultural stigma influences their peers’ willingness to discuss mental health issues and seek help, as well as the level of support provided by their families. These findings highlight the importance of raising family awareness to support the well-being of youth, as well as the necessity of creating support structures within institutions, especially educational ones. Policy makers are recommended to design strategies to improve citizen awareness and to further reform health policies in these regions by focusing more on prevention. This would contribute to reducing stigma and increasing mental health literacy, thereby providing greater support to youth from family and community (49). Simultaneously, the development and integration of peer-to-peer programs, particularly within educational institutions, would advance social support among youth. Peer-to-peer interventions, such as mutual support groups that are easily integrated into educational systems, have shown efficacy in reducing stigma and improving intentions to seek help (4). Additionally, digital interventions can be considered as cost-effective ways to expand the range of available services for youth (50). Both these initiatives can also facilitate the use of professional services, which should be integrated within educational systems (51).

University counseling centers are crucial in addressing the mental health needs of university students and are increasingly recognized as essential in shaping the overall mental health landscape within academic institutions. These centers not only address immediate mental health needs but also engage in community-building and educational efforts that inform and transform the institutional culture regarding mental health (52). Improving educational strategies and training academic staff with a focus on the holistic support of students and integrating well-being components into pedagogical processes should also be considered (4). Feedback from students across all three countries highlights notable gaps in mental health services within universities, exacerbated by a lack of faculty engagement and understanding.

The effectiveness of community engagement in addressing mental health largely depends on broader societal attitudes and legal frameworks. Saraceno and Saxena argue that societal norms greatly affect how available and effective mental health interventions are (38). When societies are reluctant to openly discuss mental health issues, this leads to sporadic community initiatives and underfunded programs. This highlights the need for societal changes to create a more supportive community environment that can sustain and improve mental health initiatives, as seen in developed countries with their comprehensive mental health policies (49, 53).

The current findings also underscore the necessity for community mental health services across Kosovo, North Macedonia, and Albania, as well as the need to improve the quality of existing services to effectively meet community needs. In advancing the quality of these services, it is particularly important to measure and develop mental health programs in a manner that is culturally sensitive while simultaneously enhancing cultural competence training for healthcare providers (15, 16). The involvement of youth in designing supportive services within the community and the educational institutions is also crucial and impactful in improving their attitudes toward seeking help (51). Furthermore, continuous and integrated initiatives within communities are critical, as opposed to sporadic or one-off campaigns. This approach will help establish a more consistent and reliable support system that can significantly improve the mental health landscape of these regions.

The intricate relationships among these levels suggest that interventions need to be multifaceted and synchronized across the socio-ecological spectrum to be effective. By increasing family awareness, enhancing education programs at the organizational level, and concurrently working to shift community and societal perceptions through, among others, policy reformation, these countries can foster an environment that promotes both individual well-being and public health. Increasing professional availability and enhancing collaboration within health agencies, non-governmental and community organizations, and educational institutions can further promote and protect the well-being of all citizens, particularly youth. Additionally, increasing collaboration with international initiatives, strengthening research partnerships to facilitate knowledge exchange, and standardizing measures to assess needs and establishing monitoring mechanisms for the quality of services should be considered opportunities for creating suitable and sustainable programs (54). By working together, these entities can effectively address the mental health needs of the population, ensuring a comprehensive approach to mental well-being (54).

### 4.1 Limitations and future research directions

While our study provides critical insights into attitudes toward mental health among university students, the qualitative design and the focus group methodology present some limitations. Focus groups, though valuable for gaining depth of insight, may not capture the full diversity of student experiences and perspectives owing to the limited number of participants and potential for self-selection bias. This limitation highlights the need for complementing focus groups with surveys or interviews across a larger sample, which can provide a broader understanding of the generalizability of our findings. Additionally, integrating a quantitative component to measure the prevalence of attitudes and experiences could enrich the comprehensiveness of the current results.

Another significant limitation is the lack of comparative analysis based on specific socio-economic factors, sex, sexual orientation, field of study, and year of study. These variables can deeply influence mental health perceptions and the effectiveness of support systems. Additionally, it is important to note that the study sample was drawn exclusively from Albanian communities within Kosovo, North Macedonia, and Albania. This presents a limitation in terms of the representativeness of the findings for other ethnicities within the studied countries. Consequently, the insights gained may not be fully applicable or reflective of the broader youth populations, particularly those from non-Albanian ethnic or cultural backgrounds. To address this limitation, future studies should aim to include a more ethnically diverse sample that encompasses various cultural and ethnic groups within these regions. Stratifying data according to socio-economic factors and student characteristics is also crucial to understand how different groups within the university population experience mental health issues and access support systems. Previous research from other countries suggests that mental health awareness and help-seeking behaviors can differ significantly across different groups of students from various subcultures, ethnicities, religions, and other demographics (2, 55–58).

## 5 Conclusion

Integrating the socio-ecological model with an understanding of the specific historical and cultural context of Kosovo, North Macedonia and Albania enabled a comprehensive analysis of the mental health stigma, challenges, and opportunities available to Albanian students in these countries. Our study indicated the necessity for a holistic approach that considers the unique dynamics of each level of influence, from the individual to the societal, to effectively combat stigma and enhance mental health support systems among this group of population. By addressing these interconnected factors, there is potential to create more robust and culturally attuned mental health services than can significantly improve public health outcomes in these regions.

## Data Availability

The data that support the findings of this study are available from corresponding author, upon reasonable request.
